# Supplementary material for: Lateral presentation of faces alters overall viewing strategy
Source: PeerJ. 2016 Jul 19;4:e2241. doi: 10.7717/peerj.2241 (PMC4958001; doi:10.7717/peerj.2241)
Supplement: Supplemental Information 1 [file peerj-04-2241-s001.docx]

Raw data and analysis code.

Luke, Chris (2016): Lateral Presentation Alters Overall Viewing Strategy. figshare.

https://dx.doi.org/10.6084/m9.figshare.3126766.v1

Includes raw data from the TobiiT60 XL eye tracker and behavioural data for each participant as well as Matlab analysis code.

To view the region of interest maps used for analysis please navigate to the following link:

Luke, Chris (2016): Lateral Presentation Alters Overall Viewing Strategy Regions of Interest. figshare.

[**https://dx.doi.org/10.6084/m9.figshare.3381166.v2**](https://dx.doi.org/10.6084/m9.figshare.3381166.v2)

Retrieved: 09 21, Jun 01, 2016 (GMT)
